# Identification and Functional Analysis of Tartrate-Resistant Acid Phosphatase Type 5b (TRAP5b) in *Oreochromis niloticus*

**DOI:** 10.3390/ijms24087179

**Published:** 2023-04-13

**Authors:** Yang Lei, Shengli Fu, Yanjian Yang, Jianlin Chen, Bingxi Li, Zheng Guo, Jianmin Ye

**Affiliations:** 1 Guangzhou Key Laboratory of Subtropical Biodiversity and Biomonitoring, Institute of Modern Aquaculture Science and Engineering, School of Life Sciences, South China Normal University, Guangzhou 510631, China; 2 Guangdong Provincial Engineering Technology Research Center for Environmentally-Friendly Aquaculture, South China Normal University, Guangzhou 510631, China

**Keywords:** tartrate-resistant acid phosphatase type 5b (TRAP5b), *Oreochromis niloticus*, phosphatase activity, reactive oxygen species, phagocytosis

## Abstract

Tartrate-resistant acid phosphatase type 5 (TRAP5) is an enzyme that is highly expressed in activated macrophages and osteoclasts and plays important biological functions in mammalian immune defense systems. In the study, we investigated the functions of tartrate-resistant acid phosphatase type 5b from *Oreochromis niloticus* (OnTRAP5b). The OnTRAP5b gene has an open reading frame of 975 bp, which encodes a mature peptide consisting of 302 amino acids with a molecular weight of 33.448 kDa. The OnTRAP5b protein contains a metallophosphatase domain with metal binding and active sites. Phylogenetic analysis revealed that OnTRAP5b is clustered with TRAP5b of teleost fish and shares a high amino acid sequence similarity with other TRAP5b in teleost fish (61.73–98.15%). Tissues expression analysis showed that OnTRAP5b was most abundant in the liver and was also widely expressed in other tissues. Upon challenge with *Streptococcus agalactiae* and *Aeromonas hydrophila* in vivo and in vitro, the expression of OnTRAP5b was significantly up-regulated. Additionally, the purified recombinant OnTRAP5b ((r)OnTRAP5) protein exhibited optimal phosphatase activity at pH 5.0 and an ideal temperature of 50 °C. The *V*_max_, *K*_m,_ and *k*_cat_ of purified (r)OnTRAP5b were found to be 0.484 μmol × min^−1^ × mg^−1^, 2.112 mM, and 0.27 s^−1^ with respect to *p*NPP as a substrate, respectively. Its phosphatase activity was differentially affected by metal ions (K^+^, Na^+^, Mg^2+^, Ca^2+^, Mn^2+^, Cu^2+^, Zn^2+^, and Fe^3+^) and inhibitors (sodium tartrate, sodium fluoride, and EDTA). Furthermore, (r)OnTRAP5b was found to promote the expression of inflammatory-related genes in head kidney macrophages and induce reactive oxygen expression and phagocytosis. Moreover, OnTRAP5b overexpression and knockdown had a significant effect on bacterial proliferation in vivo. When taken together, our findings suggest that OnTRAP5b plays a significant role in the immune response against bacterial infection in Nile tilapia.

## 1. Introduction

Tartrate-resistant acid phosphatase type 5 (TRAP5, ACP5, EC 3.1.3.2) belongs to the purple acid phosphatase family and exhibits significant tolerance to tartrate inhibition [1]. TRAP5 is widely distributed across various species, including plants [2,3], animals [4], bacteria, and fungi [5,6,7]. TRAP5 is expressed in a diverse range of cells, including osteoclasts, macrophages, dendritic cells (DCs), and tissues such as the spleen, bone, lung, and placenta [8,9,10]. In adult mice, TRAP5 is expressed extensively in various tissues [9,10,11,12]. TRAP5 has been isolated from other mammals, such as the bovine spleen [13], the spleen of patients with hairy cell leukemia [14] and Gaucher’s disease [15], human bone [16], lungs [17], and placenta [18]. In mammals, the binuclear iron core of TRAP5 contains two iron elements, one of which is stable and coupled with tyrosine residues, while the other is active in oxidation [19]. However, the binuclear metal center varies between species, with plants possessing Fe^2+^/Fe^3+^, Mn^2+^/Fe^3+^, or Zn^2+^/Fe^3+^ [12,20,21]. The binuclear iron core of the TRAP5′s molecular structure is crucial for the enzyme’s function.

TRAP5 is a regulator of bone resorption and osteoblast metabolic function, exerting its effects through phosphatase activity and reactive oxygen species (ROS) production [22,23]. In human DC, LPS has been shown to promote TRAP5 activity, suggesting a potential role for TRAP5 in immune response [10]. Overexpressing of TRAP5 in a macrophage-like cell line led to increased ROS production, indicating involvement in the inflammatory response and pathogen clearance [24]. TRAP5 knockout mice exhibited significantly delayed clearance of the microbial pathogen *Staphylococcus aureus* when challenged in vivo [25]. In addition, TRAP5 knockout in macrophages affected recruitment to pathogen bacteria, and TRAP5 was found to colocalize with the late endosomal/lysosomal marker and *S. aureus* [26]. When taken together, these findings suggest that TRAP5 plays a role in the inflammatory response.

Currently, there is limited knowledge regarding the role of TRAP5 in teleost fish in response to immune challenges and bacterial infections. Nile tilapia is a commercially important fish species that is susceptible to pathogenic bacterial infections during breeding. Therefore, it is essential to investigate the innate immune system of Nile tilapia. The Nile tilapia transcriptome database contains two types, TRAP5a, and TRAP5b. TRAP5a has three distinct isoforms, while the mRNA sequence of TRAP5b is specific. In this study, we cloned the TRAP5b homolog in Nile tilapia, designated as OnTRAP5b, and analyzed its tissue expression patterns. We also examined the changes in OnTRAP5b expression following stimulation with *S. agalactiae* and *A. hydrophila* in vivo and in vitro. Additionally, we evaluated the phosphatase activity of prokaryotic recombinant protein (r)OnTRAP5b and its response to various metal ions and inhibitors. Furthermore, we investigated the immune-related molecules, ROS, and phagocytosis of head kidney macrophages (MΦ) in the presence of (r)OnTRAP5b. Moreover, we demonstrated that OnTRAP5b significantly influenced bacterial loads in different tissues using overexpression and RNAi technology. These findings provide insights into the functional role of OnTRAP5b in teleost fish.

## 2. Results

### 2.1. Molecular Characterization of OnTRAP5b

**Figure 1:**
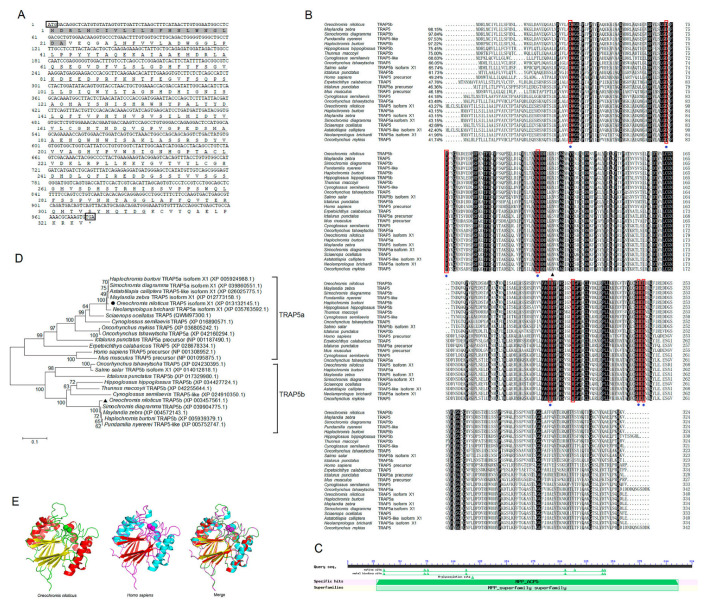
Structure and sequence analysis of TRAP5b from Nile tilapia. (**A**) The nucleotide and deduced amino acid sequences of OnTRAP5b. The signal peptide is marked in a gray shadow. The metallophosphatase domain is marked with a single underline. The translation start (ATG) and stop (TGA) codons are boxed. (**B**,**C**) Alignment of the predicted amino acid sequences of OnTRAP5b homologs and predicted conserved domains of OnTRAP5b. Symbol (●) represents metal binding sites, and active sites are in red boxed. Symbol (▲) represents the N-glycosylation site. The numbers indicate the sequence identities between OnTRAP5b and the compared sequences. The conserved and identical residues are represented by black shading, and the residues that are ≥75% identical among the aligned are in gray. (**D**) Phylogenetic tree analysis of OnTRAP5b among other vertebrates. A neighbor-joining phylogenetic tree of TRAP5 proteins based on protein sequence analyzed with mega 6.0. The degree of confidence in each branch point was determined by bootstrap analysis (1000 times). (**E**) The predicted three-dimensional structure of protein TRAP5b from *Oreochromis niloticus* (**left**), *Homo sapiens* (**middle**), and overlapped three-dimensional structure of TRAP5 from them (**right**).

The open reading frame (ORF) of OnTRAP5b was found to be 975 bp in length, which is predicted to encode a protein consisting of 324 amino acids. The predicted signal peptide of OnTRAP5b contained 22 amino acid residues. The mature peptide of OnTRAP5b was estimated to have a molecular weight of 33.448 kDa. The NCBI conserved domains program prediction indicated that the metallophosphatase (MPP) domain of OnTRAP5b is located between the 28th and 311th amino acid residues (Figure 1A). Multiple sequence alignment revealed that the sequence similarity of OnTRAP5b with other teleost TRAP5b ranged from 61.73% to 98.15%, with the highest (98.15%) being with *Maylandia zebra* and the lowest (61.73%) with *Ictalurus punctatus.* OnTRAP5b shared only 41.74 to 49.24% of the similarities of other species TRAP5a (Figure 1B). The MPP-conserved domain of OnTRAP5b was found to contain one N-glycosylation site, seven metal binding sites, and eight active sites (Figure 1C). Phylogenetic analysis revealed that TRAP5 has two major branches, TRAP5a and TRAP5b, and OnTRAP5b belongs to the member of the fish TRAP5b group and is closely related to TRAP5 of *Haplochromis burtoni*, *Pundamilia nyererei*, *Simochromis diagramma* and *M. zebra* (Figure 1D). The three-dimensional structure of OnTRAP5b was predicted by SWISS-MODEL and was found to be similar in shape to that of TRAP5 of *Homo sapiens* (Figure 1E). In NCBI, there are two types of tartrate-resistant acid phosphatase gene sequences from *O. niloticus* that could be queried, respectively defined as ACP5a (TRAP5a) and ACP5b (TRAP5b). ACP5a occurs as three distinct isoforms, namely TRAP5 isoform X1, TRAP5 isoform X2, and TRAP5 isoform X3. ACP5b has no isoforms, and the mRNA sequence of ACP5b is unique. Three distinct isoforms are completely similar except for the length difference of the N-terminal sequence. Sequence alignment of TRAP5 isoform X1 and TRAP5b exhibits a similarity of 42.27%.

### 2.2. Tissue Distribution Analysis of OnTRAP5b

**Figure 2:**
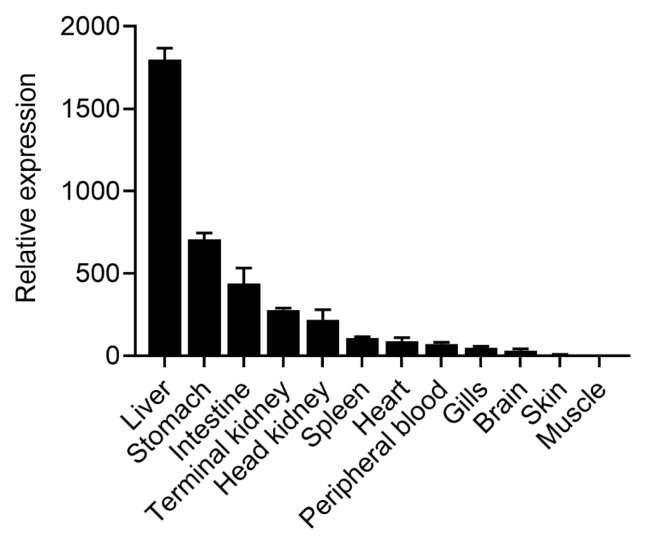
Expression analysis of OnTRAP5b in different tissues under normal physiological conditions. qRT-PCR was performed with cDNA samples prepared from the liver, stomach, intestine, terminal kidney, head kidney, spleen, heart, peripheral blood, gills, brain, skin, and muscle. The expression level in the muscle with the lowest expression was set as 1, and target gene expression was normalized against an endogenous control (*β*-actin). The data are presented as mean ± SD (*n* = 3). *n*, the number of times the experiments were performed.

In this study, qRT-PCR was used to evaluate the tissue distribution and relative expression of OnTRAP5b in healthy tilapia. The results indicated that OnTRAP5b mRNA was most highly expressed in the liver (1797.83 ± 69.86-fold higher), followed by the stomach (708.06 ± 37.44-fold higher), intestine (437.94 ± 37.44-fold higher), terminal kidney (278.91 ± 11.73-fold higher), head kidney (218.95 ± 60.77-fold higher), spleen (108.95 ± 8.02-fold higher), heart (89.63 ± 21.59-fold higher), peripheral blood (72.88 ± 10.08-fold higher), gills (49.40 ± 9.12-fold higher), brain (32.00 ± 11.18-fold higher), and skin (8.74 ± 1.05-fold higher). Conversely, OnTRAP5b expression was lowest in muscle (set as 1) (Figure 2).

### 2.3. Expression Pattern of OnTRAP5b upon S. agalactiae and A. hydrophila Challenged In Vivo

**Figure 3:**
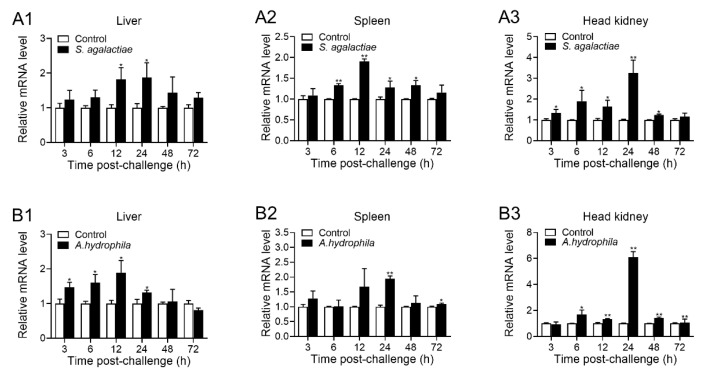
Expression pattern of OnTRAP5b in vivo. The mRNA expression of OnTRAP5b after injection with *S. agalactiae* (**A1**–**A3**) and *A. hydrophila* (**B1**–**B3**) in the liver, spleen, and head kidney at various time points. Expression values are normalized against *β*-actin, and the folding unit was calculated, deciding the values of the vaccinated tissues by PBS. The values are shown as means ± SD (*n* = 3), *n*, the number of times the experiments were performed. ** *p* < 0.01, * *p* < 0.05.

Upon stimulation with *S. agalactiae* or *A. hydrophila*, the expression of OnTRAP5b was significantly elevated in the liver, spleen, and head kidney compared to the control group at 3, 6, 12, 24, 48, and 72 h post-infection (h p.i.) (Figure 3). Following the challenge with *S. agalactiae*, the peak expression (1.88-fold increase) was observed at 24 h p.i. in the liver (Figure 3(A1)), 1.91-fold increase at 12 h p.i. in the spleen (Figure 3(A2)), and 3.25-fold increase reached at 24 h p.i in the head kidney (Figure 3(A3)). When infected with *A. hydrophila*, the peak induction occurred at 12 h p.i. in the liver (1.89-fold increase) (Figure 3(B1)), 1.94-fold increase at 24 h p.i. in the spleen (Figure 3(B2)), and 6.10-fold increase at 24 h p.i. in the head kidney (Figure 3(B3)).

### 2.4. Expression of OnTRAP5b after Stimulation In Vitro

**Figure 4:**
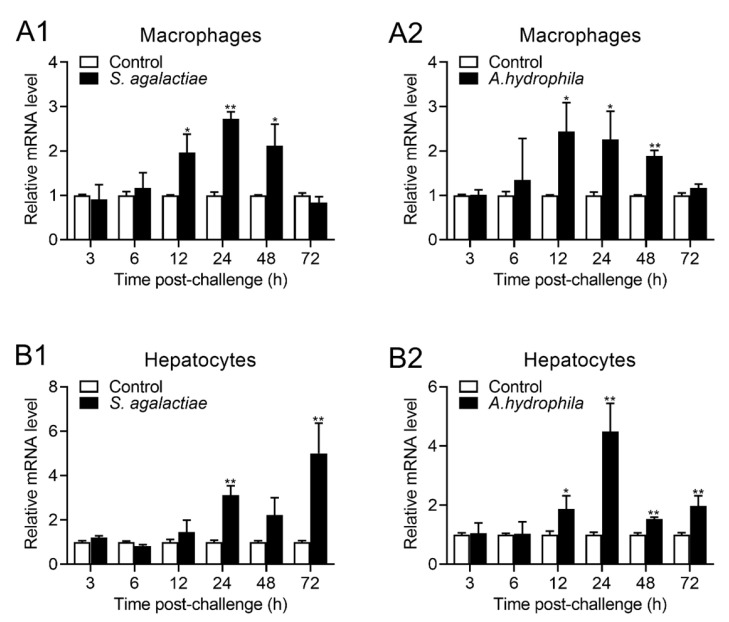
The expression pattern of OnTRAP5b in vitro. The mRNA expression of OnTRAP5b in head kidney macrophages (**A1**,**A2**) and the hepatocytes (**B1**,**B2**) after treatment with *S. agalactiae* (1 × 10^8^ CFU/mL), *A. hydrophila* (1 × 10^8^ CFU/mL) and a control group treated with PBS. The mRNA level of the OnTRAP5b gene was normalized to *β*-actin, and the fold units were calculated, deciding the value of the PBS-treated cells. The values are shown as means ± SD (*n* = 3), *n*, the number of times the experiments were performed. ** *p* < 0.01, * *p* < 0.05.

In order to investigate the impact of stimuli on the expression of OnTRAP5b in vitro, isolated MΦ and hepatocytes were stimulated with *S. agalactiae* and *A. hydrophila*. The highest expression of OnTRAP5b in MΦ occurred at 24 h p.i. with *S. agalactiae* challenge, resulting in a 2.75-fold increase (Figure 4(A1)). Similarly, the highest expression of OnTRAP5b in MΦ occurred at 12 h p.i. after the *A. hydrophila* challenge, resulting in a 2.44-fold increase (Figure 4(A2)). In hepatocytes, OnTRAP5b expression peaked at 24 h p.i with the challenge of *S. agalactiae* (3.12-fold increase) (Figure 4(B1)) and at 24 h p.i when challenged with *A. hydrophila* (4.49-fold increase) (Figure 4(B2)).

### 2.5. Purification and Phosphatase Activity of Recombinant (r)OnTRAP5b

The recombinant protein (r)OnTRAP5b and (r)MBP were expressed in *E. coli* K12 strains TB1 and subsequently purified using an amylose resin column. SDS-PAGE analysis confirmed the presence of a single band with the expected molecular mass for both (r)OnTRAP5b and (r)MBP (Figure S1).

**Figure 5:**
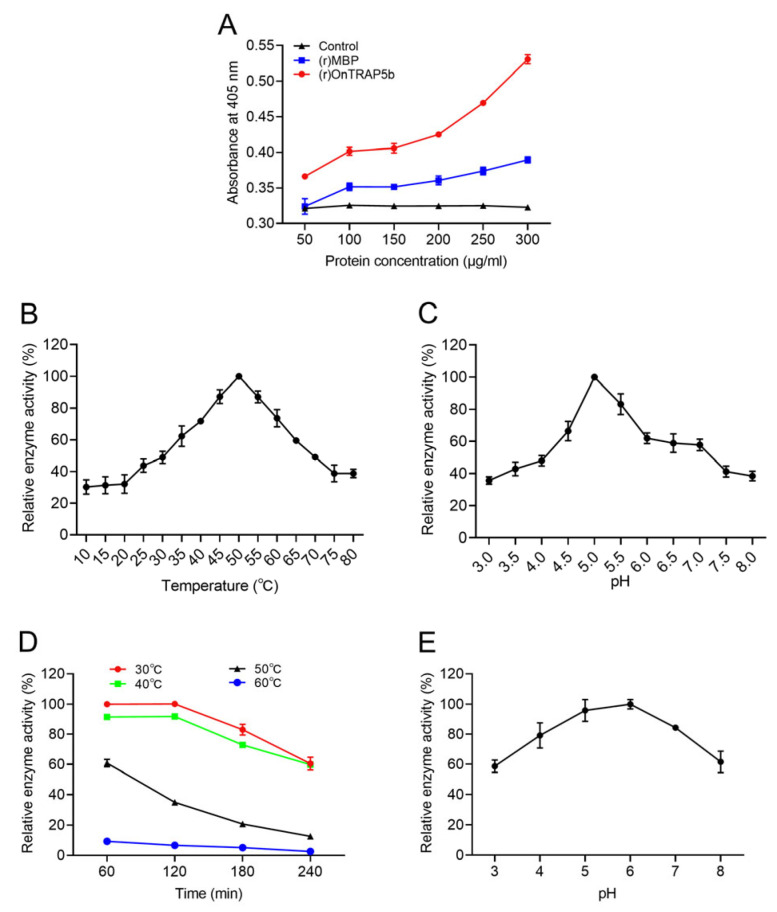
Phosphatase activity of (r)OnTRAP5b. Analysis dependence on protein concentration (**A**); optimum temperature (**B**); optimum pH (**C**); temperature stability (**D**); and pH stability (**E**) of (r)OnTRAP5b. Phosphatase activity of (r)OnTRAP5b were expressed as percentages of the relative phosphatase activity. Data are presented as means ± SD (*n* = 3). *n*, the number of times the experiments were performed.

In order to assess the phosphatase activity of (r)OnTRAP5b, varying concentrations of the recombinant protein (r)OnTRAP5b (50–300 μg/mL) and (r)MBP (50–300 μg/mL) were incubated with substrate *p*NPP (5 mM) in buffer at 37 °C for 30 min (Figure 5A). The measured absorbance value of the (r)OnTRAP5b group, which ranged from 0.366–0.531 when hydrolyzing *p*NPP with (r)OnTRAP5b at 50–300 μg/mL, was significantly higher than that of (r)MBP (0.324–0.390). While the absorbance value of the PBS control group was 0.321–0.325. In order to investigate the effect of temperature and pH on (r)OnTRAP5b phosphatase activity, the temperature was varied from 10–80 °C and the pH from 3–8. The optimal reaction temperature for (r)OnTRAP5b was found to be 50 °C (Figure 5B), and the optimal pH was 5.0 (Figure 5C). The *V*_max_, *K*_m_, and *k*_cat_ values for (r)OnTRAP5b using *p*NPP as substrate were determined to be 0.484 μmol × min^−1^ × mg^−1^, 2.112 mM, and 0.27 s^−1^, respectively.

The thermal stability analysis of (r)OnTRAP5b demonstrated its relative stability when subjected to incubation at 30 or 40 °C for 120 min (Figure 5D). The residual phosphatase activity of (r)OnTRAP5b was determined after incubation at 30 °C for 60 min, and this value was set as the maximum relative phosphatase activity (100%). Subsequent incubation at 30 °C for 180 min and 240 min resulted in residual phosphatase activities of 82.99% and 60.40%, respectively. Similarly, incubation at 40 °C for 180 min and 240 min resulted in residual phosphatase activity of 72.82% and 59.77%, respectively. At 50 °C, the phosphatase activity of (r)OnTRAP5b was 60.79% after 60 min and 12.58% after 240 min. At 60 °C, the phosphatase activity of (r)OnTRAP5b was only 9.26–2.57% for 60 to 240 min.

The stability of (r)OnTRAP5b was evaluated by incubating it overnight at 4 °C in buffer solution with pH values ranging from 3.0 to 8.0, and the residual phosphatase activity was determined at the optimal pH and temperature of 37 °C. (r)OnTRAP5b was found to be relatively stable between pH 4.0–7.0, with more than 70% residual phosphatase activity (Figure 5E).

### 2.6. Effect of Metal Ions and Inhibitors on (r)OnTRAP5b Phosphatase Activity

**Figure 6:**
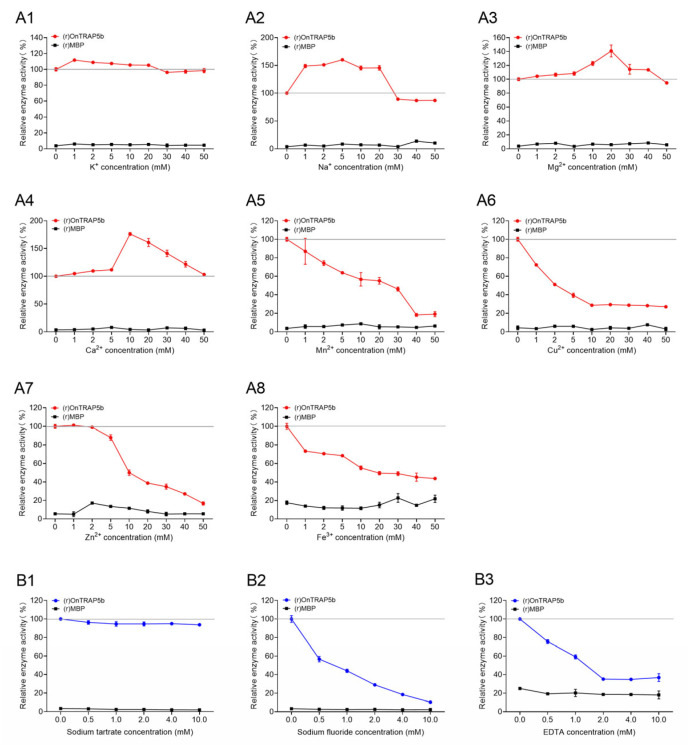
Effects of different metal ions and different inhibitors on (r)OnTRAP5b. The enzyme activities of (r)OnTRAP5b under different metal ions (**A1**–**A8**) and different inhibitors (**B1**–**B3**) were expressed as percentages of the relative phosphatase activity. Data are presented as means ± SD (*n* = 3). *n*, the number of times the experiments were performed.

The impact of metal ions with varying concentrations on the phosphatase activity of (r)OnTRAP5b was investigated. The metal ions K^+^, Na^+^, Mg^2+^, Ca^2+^, Mn^2+^, Cu^2+^, Zn^2+^, and Fe^3+^ were found to significantly affect the phosphatase activity of (r)OnTRAP5b. The control for this study was the phosphatase activity of (r)OnTRAP5b without metal ions, which was set as 100%. The phosphatase activity measured with 1–50 mM of different metal ions was considered relative enzyme activity. K^+^, Na^+^, Ca^2+^, and Mg^2+^ were found to enhance the (r)OnTRAP5b phosphatase activity in a concentration-dependent manner within a certain range (Figure 6(A1–A4)). Conversely, Zn^2+^, Fe^3+^, Mn^2+^, and Cu^2+^ were found to inhibit its phosphatase activity in a concentration-dependent manner within a certain range (Figure 6(A5–A8)). The effect of these metal ions on the enzyme tended to stabilize when their concentration increased to a certain extent. In contrast, (r)MBP did not exhibit any relative phosphatase activity in the 0–50 mM range of different metal ions.

The experiments depicted in Figure 6 were conducted to assess the sensitivity of (r)OnTRAP5b to three inhibitors. The control for the phosphatase activity of (r)OnTRAP5b without inhibitors was established as 100%, and the relative enzyme activities were determined for phosphatase activity measured with 0.5–10 mM of different inhibitors. Figure 6(B1) revealed that sodium tartrate had no significant effect on phosphatase activity within a limited range. However, the phosphatase activity of (r)OnTRAP5b was inhibited by sodium fluoride or EDTA in a concentration-dependent manner within a certain extent (0.5–10 mM) (Figure 6(B2,B3)). Furthermore, (r)MBP exhibited no phosphatase activity in the range of 0.5–10 mM for sodium tartrate, sodium fluoride, and EDTA.

### 2.7. Effects of (r)OnTRAP5b on mRNA Expression of Inflammatory-Related Cytokines of Tilapia Head Kidney Macrophages

**Figure 7:**
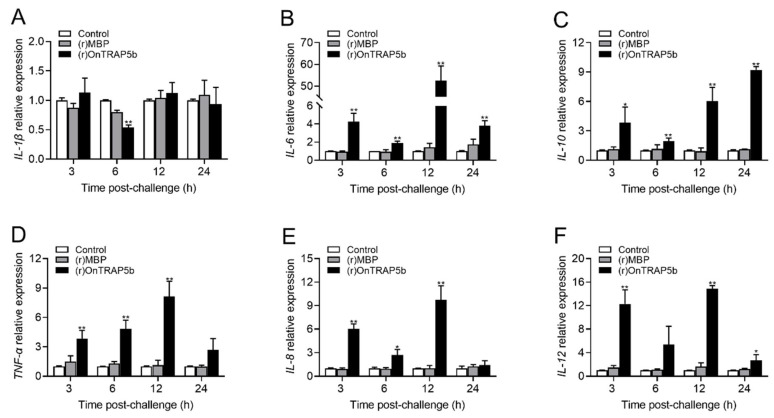
Analysis of the expression of immune-related genes in head kidney macrophages. Nile tilapia head kidney macrophages were treated with (r)OnTRAP5b (100 μg/mL), (r)MBP (100 μg/mL), and PBS. After 3, 6, 12, and 24 h of stimulation, the expression of *IL-1β* (**A**); *IL-6* (**B**); *IL-10* (**C**); *TNF-α* (**D**); *IL-8* (**E**); and *IL-12* (**F**) were examined. The expression of the six genes was normalized against *β*-actin, and the expression of these six immune genes in PBS-treated cells was set to 1. The values are shown as means ± SD (*n* = 3), *n*, the number of times the experiments were performed. ** *p* < 0.01, * *p* < 0.05.

The expression levels of several inflammatory-related cytokines were measured in MΦ treated with (r)OnTRAP5b (100 μg/mL) using qRT-PCR. The results demonstrated a significant increase in mRNA expression of *IL-1β*, *IL-6*, *IL-10*, *TNF-α*, *IL-8*, and *IL-12* in MΦ. The expression of *IL-1β* was increased compared to the control group at 3 (1.13-fold ± 0.25-fold higher) and 12 h (1.12 ± 0.18-fold higher), while down-regulated at 6 h (0.54 ± 0.04-fold) and not change at 24 h (0.94 ± 0.28-fold). The expression level of *IL-1β* was not statistically significant when compared to the control group at the three-time points of 3, 12, and 24 h. (Figure 7A) The expression of *IL-6* (~52.53-fold) (Figure 7B), *TNF-α* (~8.14-fold) (Figure 7D), *IL-8* (~9.64-fold) (Figure 7E), and *IL-12* (~14.91-fold) (Figure 7F) peaked at 12 h after treatment with (r)OnTRAP5b. The expression of *IL-10* was significantly increased at 3 (~3.84-fold), 6 (~1.95-fold), 12 (~6.03-fold), and 24 h (~9.21-fold) (Figure 7C). In comparison to the control group, (r)MBP (100 μg/mL) had no significant impact on the expression of these inflammatory-related cytokines.

### 2.8. Effects of (r)OnTRAP5b on the Production of ROS and Phagocytic Activity in MΦ

**Figure 8:**
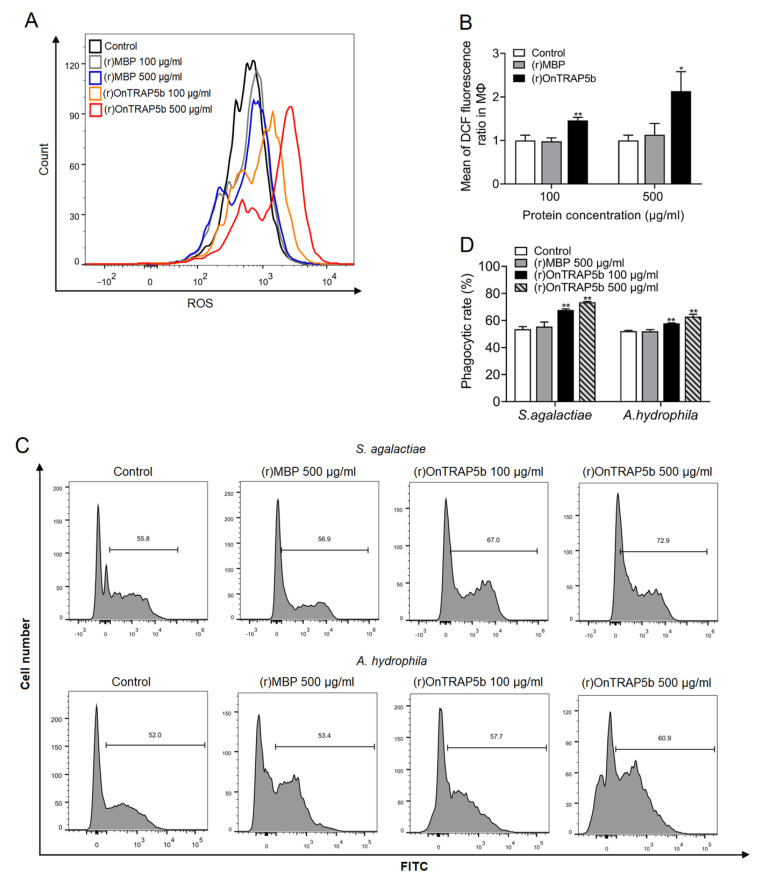
The analysis of ROS production and phagocytic activities in head kidney macrophages upon (r)OnTRAP5b. Head kidney macrophages were stimulated with 100 or 500 μg/mL (r)OnTRAP5b or (r)MBP, and oxidation of DCFH-DA was measured for 30 min using excitation and emission filters of 488 and 525 nm, respectively (**A**). The mean of DCFH-DA fluorescence with treatment for (r)OnTRAP5b or (r)MBP at doses of 100 and 500 μg/mL in head kidney macrophages (**B**). Histogram distribution of uptake *S. agalactiae* and *A. hydrophila* in head kidney macrophages with treatment for (r)OnTRAP5b or (r)MBP at doses of 100 and 500 μg/mL (**C**). The phagocytic rate of uptake *S. agalactiae* and *A. hydrophila* in head kidney macrophages with treatment for (r)OnTRAP5b or (r)MBP at doses of 100 and 500 μg/mL (**D**). The values are shown as means ± SD (*n* = 3), *n*, the number of times the experiments were performed. ** *p* < 0.01, * *p* < 0.05.

In order to analyze the viability of macrophages treated with various doses of (r)OnTRAP5b, the concentrations of (r)OnTRAP5b and (r)MBP ranging from 100–500 μg/mL were investigated and were found to have no significant effect on the viability of MΦ compared to the control group (Figure S2). The production of ROS was significantly increased in association with stimulation of (r)OnTRAP5b at 100 μg/mL and 500 μg/mL. The intracellular oxidation status of MΦ was measured with DCFH-DA by flow cytometry (Figure 8A). Statistical analysis showed that (r)OnTRAP5b at 100 μg/mL and 500 μg/mL significantly increased ROS production by 1.46-fold and 2.13-fold, respectively (Figure 8B). Treatment of cells with (r)MBP had no noticeable effect on ROS production. The phagocytic activities of MΦ were analyzed by incubating them with *S. agalactiae* and *A. hydrophila* for flow cytometry analysis to determine whether treatment with (r)OnTRAP5b could enhance phagocytosis. The results showed that (r)OnTRAP5b could considerably increase MΦ phagocytosis (Figure 8C). The phagocytic rate of *S. agalactiae* and *A. hydrophila* in MΦ were significantly increased after treatment with (r)OnTRAP5b at the dose of 100 μg/mL and 500 μg/mL (Figure 8D). Treatment of MΦ with (r)MBP or PBS had no significant effect on phagocytic activity.

### 2.9. Effect of OnTRAP5b Overexpression and Knockdown on Bacteria Load In Vivo

**Figure 9:**
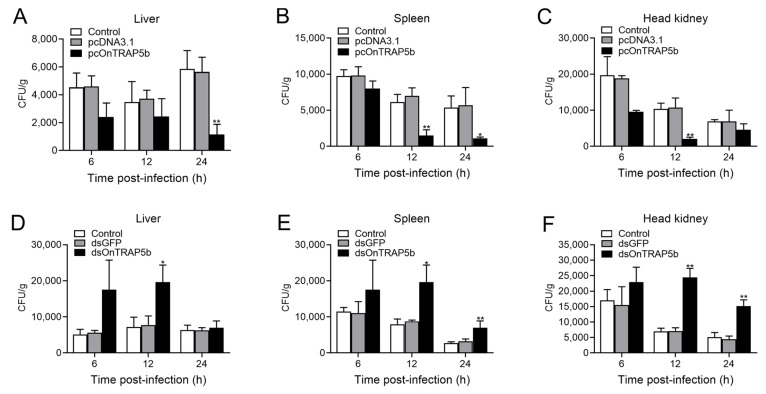
Analysis of the effects of overexpression and knockdown expression of OnTRAP5b on *S. agalactiae* infection. Nile tilapia administered with pcOnTRAP5b, pcDNA3.1, dsOnTRAP5b, dsGFP, or PBS (control) were infected with *S. agalactiae*. Bacterial loads in the liver (**A**,**D**), spleen (**B**,**E**), and head kidney (**C**,**F**) at 6, 12, and 24 h after *S. agalactiae* infection were determined. The values are shown as means ± SD (*n* = 3), *n*, the number of times the experiments were performed. ** *p* < 0.01, * *p* < 0.05.

Overexpression of OnTRAP5b resulted in increased expression of OnTRAP5b in the liver (~1.42-fold), spleen (~1.67-fold), and head kidney (~1.67-fold) in the pcOnTRAP5b-treated group compared to the control group, as determined by qRT-PCR (Figure S3A–C). Conversely, the relative mRNA level of OnTRAP5b did not significantly differ between the pcDNA3.1-treated group and the control group. Bacterial load in the liver, spleen, and head kidney were determined at 6, 12, and 24 h p.i using plate counting. At 24 h p.i., pcOnTRAP5b-treated fish exhibited a significantly lower bacterial load in the liver (4.65-fold) (Figure 9A). The number of bacterial colonies in the spleen (Figure 9B) of pcOnTRAP5b-treated fish was significantly reduced at 12 and 24 h p.i. (~4.10-fold and ~4.89-fold respectively). In the head kidney, bacterial counts were lower in pcOnTRAP5b-treated fish at 6, 12, and 24 h p.i. (~2.07-fold, ~5.14-fold and ~1.50-fold respectively) (Figure 9C). In contrast, there were no significant differences in bacterial colony number between the pcDNA3.1-administrated and the control group.

To investigate the role of OnTRAP5b in bacterial infection, in vivo knockdown of OnTRAP5b was achieved using siRNA. At 12 h post-dsRNA injection, qRT-PCR analysis revealed a significant reduction in OnTRAP5b expression in the liver (Figure S3D), spleen (Figure S3E), and head kidney (Figure S3F) of treated fish compared to control fish (PBS). The expression of OnTRAP5b in the dsGFP-treated group was not substantially different from the control group. Fish treated with dsOnTRAP5b exhibited increased bacterial colony counts in the liver at 6, 12, and 24 h p.i. (~3.44-fold, ~2.72-fold and ~1.10-fold respectively) (Figure 9D). At 12 and 24 h p.i, fish treated with dsOnTRAP5b had significantly larger bacterial loads in the spleen (~5.65-fold and ~3.36-fold, respectively) (Figure 9E). At 12 and 24 h p.i., the bacterial colony in the head kidney was considerably higher in fish treated with dsOnTRAP5b (~3.53-fold and ~2.98-fold, respectively) (Figure 9F). In contrast, there was no significant difference in the bacterial loads between the dsGFP-treated group and the control group.

## 3. Discussion

TRAP5 is a member of the purple phosphatase family of metalloenzymes, which catalyze the hydrolysis of various phosphate esters and anhydrides under acidic reaction conditions. TRAP5 plays a crucial role in the immune defense system and has been extensively studied in mammals [24]. However, research on TRAP5 in teleost fish has been limited. In this study, we cloned a teleost TRAP5b in Nile tilapia and investigated its function in the innate immune response.

Alignment with other species revealed that the amino acid sequence of OnTRAP5b is highly conserved, exhibiting an MPP domain, eight active sites, and seven metal binding sites. However, OnTRAP5b differs from mammalian TRAP5b in that it contains an N-glycosylation site, whereas mammals possess two N-glycosylation sites [27,28,29,30]. Previous research has indicated that the nature and extent of glycosylation may serve as a mechanism for regulating TRAP5 activity and function in mammals [30]. The three-dimensional structure of OnTRAP5b is also similar to that of mammalian TRAP5. A comparative analysis of the characteristics of the tartrate-resistant acid phosphatase molecule cloned from *Sciaenops ocellatus* (SoACP5) and the OnTRAP5b sequence revealed that the similarity between OnTRAP5b and SoACP5 was only 42.69%, and phylogenetic analysis demonstrated that OnTRAP5b belongs to the fish group, while SoACP5 belongs to the TRAP5a branch.

Most acid phosphatases (ACP) exhibit activity in an acidic environment and demonstrate a broad range of heat stability. The synthetic organic chemical *p*NPP is commonly used as a substrate for ACP [31,32]. The optimum pH for TRAP5 activity from *Trypanosoma cruzi* using *p*NPP as substrate was determined to be 5.0 [33]. In this study, we also analyzed the phosphatase activity of OnTRAP5b for *p*NPP since *p*NPP could be used for the quick analysis of the protein phosphatase activity under any non-standard conditions. Previous studies have also investigated the substrate specificity of acid phosphatase and the compounds used, including *p*NPP, phosphoserine (P-Ser), phosphothreonine (P-Thr), phosphotyrosine (P-Tyr), adenosine triphosphate, adenosine diphosphate, glucose-6-phosphate, glucose-1-phosphate, fructose-6-phosphate, phenyl phosphate, pyridoxal-6-phosphate, glycerophosphate and so on [33,34]. Different acid phosphatases showed high catalytic activity for *p*NPP [33,34]. The investigation of the substrate specificity of (r)OnTRAP5b needs further verification in a future study. ACP from *Artocarpus communis* maintained 50% of its activity at 55 °C [32]. When ACP from the human leukemic spleen was subjected to incubation at 80 °C for 30 min, it retained only 10% of its activity [35]. *Thermus thermophilus* ACP was found to be active within the range of 40–90 °C, with an optimum temperature of 70 °C [36]. In this study, the optimal pH for *p*NPP catalysis by OnTRAP5b was determined to be 5.0, with an optimal temperature of 50 °C. (r)OnTRAP5b was shown to be stable in the pH range of 4.0–7.0 based on acid-base stability analysis and stable in the temperature range of 30–40 °C based on temperature stability analysis. Previous research on SoACP5 revealed that the optimum pH for the catalytic activity of SoACP5 toward the substrate *p*NPP was 5.5, with an optimal temperature of 55 °C [37]. These findings suggest that OnTRAP5 and SoACP5 exhibit similar dependence on H^+^ concentration. (r)OnTRAP5b activity exhibited Michaelis-Menten behavior, with *V*_max_, *K*_m,_ and *k*_cat_ values of 0.484 μmol × min^−1^ × mg^−1^, 2.112 mM, and 0.27 s^−1^, respectively. In previous studies, *T. cruzi* tartrate-resistant acid phosphatase type 5 exhibited a *V*_max_ for *p*NPP hydrolysis of 7.7 nmol × μg^−1^ × h^−1^ and apparent *K*_m_ for *p*NPP of 169.3 μM [33]. The *K*_m_ of acid phosphatase in *Ctenopharyngodon idella* (grass carp) was 3.56 mM, and the *V*_max_ was 0.153 μmol × ml^−1^ × min^−1^ [38]. *K*_m_ and *V*_max_ of acid phosphatase from *Macrotyloma uiflorum* seeds for *p*NPP were found to be 0.934 mM and 1.333 mM× min^−1^, respectively [34].

The OnTRAP5b sequence was analyzed and found to possess a binuclear metal core. The spatial structure of a protein can be modified by combination with different metal ions, resulting in varying phosphatase activity. K^+^, Na^+^, Mg^2+^, and Ca^2+^ were found to promote the phosphatase activity of OnTRAP5b, while Mn^2+^, Cu^2+^, Zn^2+^, and Fe^3+^ downregulate phosphatase activity of (r)OnTRAP5b within a certain concentration range, similar to findings in grass carp [38]. Additionally, three inhibitors were used to detect changes in (r)OnTRAP5b phosphatase activity, with sodium tartrate having no effect, indicating that (r)OnTRAP5b belongs to the tartrate-resistant acid phosphatase family. Sodium fluoride inhibited the phosphatase activity, similar to the findings of *T. cruzi* [33]. EDTA was found to have a considerable effect on the phosphatase activity of (r)OnTRAP5b, suggesting that EDTA could chelate metal ions combined with OnTRAP5b. The effect of metal ions on phosphatase activity showed that Zn^2+^ and Fe^3+^ inhibited the activity of SoACP5 and OnTRAP5b, while Ca^2+^, Mg^2+^, and K^+^ promoted their activity [37]. These results suggest that the amino acid residues in enzyme activity can alter the conformation of protein under the influence of metal ions.

TRAP5 is a crucial factor for maintaining health, growth, metabolism, and homeostasis and is highly abundant in various tissues, including bone, spleen, liver, thymus, serum, and colon [9,24,39,40,41]. In Nile tilapia, OnTRAP5b is broadly expressed in all tissues under normal physiological conditions, with the highest expression in the liver, followed by the stomach, intestine, terminal kidney, head kidney, spleen, heart, peripheral blood, gills, brain, and skin. Upon stimulation with *S. agalactiae* and *A. hydrophila*, significant upregulation of OnTRAP5b expression was observed in vivo in the liver, spleen, and head kidney, as well as in vitro in MΦ and hepatocytes. Similarly, TRAP5 expression in the spleen and head kidney of *S. ocellatus* was dramatically up-regulated upon stimulation with *Edwardia tarda* [37]. These findings suggest that TRAP5 may play a crucial role in the immune response.

Previous research has established that TRAP5 is involved in non-specific immune responses, such as macrophage activity and inflammatory response [25,42]. In this study, (r)OnTRAP5b significantly enhanced phagocytic activity, ROS generation, and the release of inflammatory factors (*IL-1β*, *IL-6*, *IL-10*, *TNF-α*, *IL-8*, and *IL-12*) in MΦ. MΦ are essential components of the teleost professional phagocytic system, serving as one of the first lines of host defense against infections. Previous reports have demonstrated that TRAP5 can generate ROS both in vitro and in vivo. Overexpression of TRAP5 in mammalian macrophages resulted in increased intracellular ROS production with a neutral pH [43]. Furthermore, we observed that overexpression of OnTRAP5b significantly inhibited bacterial growth in vivo, whereas knockdown of OnTRAP5b promoted bacterial growth in vivo. Similarly, in mice macrophages, TRAP5 exhibited stronger bacterial killing activity against *S. aureus* after overexpression [24]. Overall, OnTRAP5b promoted macrophage activity and ROS release and played a crucial role in bacterial clearance.

In summary, the successful cloning of OnTRAP5b from Nile tilapia was achieved. OnTRAP5b was found to be expressed in a wide range of tissues. OnTRAP5b mRNA expression significantly increased in vivo and in vitro upon challenge with *S. agalactiae* and *A. hydrophila*. The phosphatase activity of (r)OnTRAP5b was affected by various metals and inhibitors. (r)OnTRAP5b was found to enhance phagocytosis, the release of ROS, and the expression of inflammatory molecules in MΦ. Overexpression or knockdown experiments demonstrated that OnTRAP5b played a crucial role in defending against pathogenic infections in vivo. These findings suggest that OnTRAP5b may be a key component of the innate immune system in Nile tilapia.

## 4. Materials and Methods

### 4.1. Fish

Nile tilapia were purchased from Guangdong Tilapia Breeding Farm (Guangzhou, China). The fish were kept in the automatic filtering aquaculture system for two weeks prior to the experiments at 28 ± 3 °C to acclimate them to the experiment environment.

### 4.2. Total RNA Isolation, cDNA Synthesis, and Real-Time Quantitative PCR

Total RNA extraction was isolated from tissues using the Trizol reagent (Vazyme, Nanjing, China) according to the manufacturer’s instructions. The RNA sample concentration was determined using NanoDrop 2000 spectrophotometer (Thermo Fisher Scientific, BioTek, Waltham, MA, USA). The first strand of cDNA was synthesized using the Hifair^®^Ⅱ 1st Strand cDNA Synthesis SuperMix according to the manufacturer’s instructions (Yeasen Biotechnology, Shanghai, China). Quantitative real-time PCR (qRT-PCR) assay did conduct with Hieff^®^ qPCR SYBR Green Master Mix (Yeasen Biotechnology, Shanghai, China).

### 4.3. Cloning and Sequence Analyses of OnTRAP5b

The complete length of the *O. niloticus* TRAP5b (OnTRAP5b) ORF sequence was cloned and confirmed by DNA sequencing (GenBank accession XM_003457513.5). OnTRAP5b specific primers (OnTRAP5b-F1/R1, Table 1) were designed using Primer Premier 5.0, and cDNA from the liver was used as the template. The primers used for OnTRAP5b preparation and analysis were specific for the mRNA encoding XP_003457561 (i.e., XM_003457513.5). The OnTRAP5b sequence was examined using the BLAST algorithm from the National Center for Biotechnology Information (NCBI, https://www.ncbi.nlm.nih.gov/pmc/ (accessed on 8 November 2021)). The location of the OnTRAP5b signal peptide site was performed using the SignalP 4.1 server (http://www.cbs.dtu.dk/services/SignalP/ (accessed on 8 November 2021)). Protein domain analysis was performed using Conserved domains (https://www.ncbi.nlm.nih.gov/Structure/cdd/wrpsb.cgi (accessed on 8 November 2021)). Multiple sequence alignments of TRAP5 amino acid sequences were performed using the DNAMAN 7.0 software. The phylogenetic relationship of OnTRAP5b was constructed using MEGA 6.0 software with 1000 bootstrap replications. The typical structures were predicted by SWISS-MODEL online software (https://swissmodel.expasy.org/interactive (accessed on 8 November 2021)) and modified by PyMOL 2.5 software.

### 4.4. OnTRAP5b Expression in Different Tissues under Normal Physiological Conditions

Twelve tissue samples (liver, stomach, intestine, terminal kidney, head kidney, spleen, heart, peripheral blood, gills, brain, skin, and muscle) were collected for qRT-PCR analysis of OnTRAP5b mRNA expression in healthy Nile tilapia. Total RNA and cDNAs were synthesized as described above. The qRT-PCR carried out on the CFX96 Touch Real-Time PCR (BioRad, Hercules, CA, USA) was applied to evaluate tissue distribution of OnTRAP5b transcription levels. The primers qOnTRAP5b-F1/R1 (Table 1) were applied to perform OnTRAP5b expression, and *β*-actin was amplified using qβ-actin-F and qβ-actin-R as internal control primers (Table 1). The 2^–ΔΔCt^ method was used to calculate the relative expression of the *OnTRAP5b* [44].

### 4.5. Expression Pattern of OnTRAP5b following S. agalactiae and A. hydrophila Stimulation In Vivo

*S. agalactiae* was cultured in brain heart infusion (BHI), and *A. hydrophila* was cultured in Luria-Bertani (LB) at 28 °C before being resuspended in sterilized phosphate-buffered saline (PBS). The experimental groups were injected with 100 μL (1 × 10^7^ Colony-Forming Units (CFU)/mL) of *S. agalactiae* or 100 μL of *A. hydrophila* intraperitoneally, while the control group was injected with 100 μL of PBS intraperitoneally [45]. At 3, 6, 12, 24, 48, and 72 h post-infection (h p.i)., liver, spleen, and head kidney samples were collected to analyze the mRNA level of OnTRAP5b. The expression levels of OnTRAP5b were measured by the 2^−ΔΔCt^ method, and *β*-actin was used as an internal control [44].

### 4.6. Isolation of Head Kidney MΦ and Hepatocytes

Head kidney MΦ from healthy *O. niloticus* (200 ± 10 g) were isolated according to a previous study [46]. In brief, the three fish’s head kidney was individually removed and placed in 5 mL Roswell Park Memorial Institute 1640 medium (RPMI-1640) (Gibco, Carlsbad, CA, USA) containing 100 U/mL penicillin G and 100 mg/mL streptomycin (P/S, Sigma-Aldrich, St. Louis, MO, USA). Then the head kidney was passed through a cell strainer to obtain cell suspension. The cell suspension was carefully layered onto an equal volume of Histonpaque^®^ 1077 (Sigma-Aldrich, MO, USA), followed by centrifugation at 500× *g* for 40 min at 4 °C. The gradient interface band of cells was collected and washed three times with RPMI-1640 medium, 1% P/S, and centrifuged at 500× *g* for 10 min at 4 °C. The cells were seeded into 96-well plates (Corning Costar Co., Waltham, MA, USA) and incubated at 25 °C for 4 h. Then, the adherent MΦ were collected and resuspended in RPMI-1640 medium containing 10% fetal bovine serum (FBS) (Gibco, CA, USA) to the concentration of 1 × 10^7^ cells/mL. The experimental group received 100 μL of formalin-inactivated *S. agalactiae* (1 × 10^8^ CFU/mL) or *A. hydrophila* (1 × 10^8^ CFU/mL). The control group was added with an equal volume of PBS. The cells were cultured at 25 °C, stimulated for 3, 6, 12, 24, 48, and 72 h, and then lysed in 1 mL of Trizol for RNA extraction.

In order to isolate Nile tilapia hepatocytes, 15 mL trypsin with 0.2% Ethylene Diamine Tetraacetic Acid (EDTA) (Gibco, Carlsbad, CA, USA) was added to each 5 g of the liver of three fish for enzymatic digestion, which was treated at 25 °C for 30 min. Following that, 2 mL of FBS was used to stop the reaction [47]. The cells were centrifuged at 500× *g*, 4 °C for 5 min and then resuspended in 3 mL of red blood cell lysis buffer (Solarbio, Beijing, China). Finally, the hepatocytes were washed in RPMI-1640 medium and suspended to the concentration of 1 × 10^7^ cells/mL in RPMI-1640 medium containing 10% FBS (Gibco, Carlsbad, CA, USA) [47]. The hepatocytes were stimulated with *S. agalactiae* (1 × 10^8^ CFU/mL) or *A. hydrophila* (1 × 10^8^ CFU/mL) and incubated in 96-well plates. The cell sample collection time was the same as that described above.

### 4.7. Plasmid Construction

In order to obtain recombinant plasmid pMD18T-OnTRAP5b, PCR products were ligated into the pMD18 T vector (TaKaRa, Dalian, China) and transformed into competent *Escherichia coli* cells Top 10. OnTRAP5b was excised from pMD18T-OnTRAP5b by digestion with *Eco*R 32I and *Bam*H I restriction enzymes (Thermo Fisher Scientific, USA). OnTRAP5b was inserted into pMAL-c5X (New England Biolabs, Ipswich, MA, USA) to produce pMAL-c5X-OnTRAP5b with an MBP-tag at N-terminus for protein purification, using the primer pair OnTRAP5b-F2/R2 (Table 1). To obtain the recombinant eukaryotic expression plasmid pcOnTRAP5b using the primer pair OnTRAP5b-F3/R3 (Table 1), the OnTRAP5b fragment was inserted into pcDNA3.1 vector at *Bam*H I and *Eco*R 32I to construct overexpression plasmid pcOnTRAP5b. The plasmid pcOnTRAP5b or pcDNA3.1 was transformed in *E. coli* Top 10 cells. Recombinant plasmids of pMD18T-OnTRAP5b, pMAL-c5X-OnTRAP5b, and pcOnTRAP5b were extracted from positive clones and confirmed by sequencing (Figure S4).

### 4.8. Expression and Purification of Recombinant Protein

The plasmids pMAL-c5X-OnTRAP5b or backbone pMAL-c5X were transformed into *E. coli* K12 strains TB1 competent cells (WeiDi, Shanghai, China) and cultured onto solid LB plates with ampicillin (100 μg/mL) at 37 °C. The culture medium containing fusion plasmid was inoculated into LB medium containing glucose and ampicillin and incubated at 37 °C to O.D._600_ to 0.6–0.8. Then isopropyl-β-D-thiogalactopyranoside (IPTG, final concentration, 0.1 mM) was added and induced at 16 °C for 16 h. The cells were harvested by centrifugation, and the supernatant was discarded and resuspended the cells in column buffer (20 mM Tris-HCl, 200 mM NaCl). After freezing at 4 °C, the cells were lysed by sonication until the amount of released protein reached a maximum, and cell debris was removed with centrifugation. The target protein was attached to the MBP-tag affinity column after purifying the protein using an amylose resin column. In order to elute the target protein, a column buffer containing maltose (10 mM) was utilized. The purified protein was stored at −80 °C until further use.

### 4.9. Phosphatase Activity of Recombinant Protein (r)OnTRAP5b

The phosphatase activity of (r)OnTRAP5b was measured by spectrophotometrically quantifying the rate of hydrolysis of 5 mM *p*-nitrophenyl phosphate (*p*NPP). Purified (r)OnTRAP5b (50–300 μg/mL) was incubated at 37 °C for 30 min in substrate buffer consisting of 50 mM Hac-NaAc (pH 5.5) and 5 mM *p*NPP. The reaction was stopped by adding 10 μL 1 M NaOH, and the absorbance at 405 nm was measured using a microplate reader of multi-wavelength (PerkinElmer, Waltham, MA, USA) [48,49]. In the control group, an equal volume of PBS was used instead of protein, and other operations were consistent with the above description. Next, the optimum pH was determined, and the substrate buffer adjusted the pH from 3.0 to 8.0. The optimal temperature was determined that the reaction was performed at 10–80 °C with optimal pH. According to the established protocol for investigating enzyme kinetics, a range of substrate concentrations (5–25 mM) was selected to determine the relationship between reaction rate and substrate concentration. The kinetic constants, including the Michaelis constant (*K*_m_) and maximum reaction rate (*V*_max_), were determined using Origin 8.0 software. These values were then combined with the molecular weight of the enzyme to calculate the catalytic number (*k*_cat_) value of the (r)OnTRAP5b, which is the number of individual reactions catalyzed by the active site per time unit when the (r)OnTRAP5b is working at *V*_max_. This approach is consistent with the previous studies in the field [50,51]. Heat stability analysis showed that 100 μg/mL (r)OnTRAP5b and (r)MBP were maintained at different temperatures (30–60 °C) for a certain time (60–240 min) and then immediately cool them. The residual phosphatase activity was measured at 37 °C with 5 mM *p*NPP. 100 μg/mL (r)OnTRAP5b and (r)MBP were kept overnight at 4 °C in buffer (pH 3.0–8.0), and residual phosphatase activity was evaluated at the optimal pH and 37 °C. The highest phosphatase activity was set as 100%, and the absorbance values measured at other temperatures and pH were converted into relative phosphatase activity.

### 4.10. Effect of Different Metal Ions, Phosphatase Specific Inhibitors or EDTA on Phosphatase Activity of (r)OnTRAP5b

To determine the effect of different metal ions (1–50 mM of KCl, NaCl, MgCl_2_, CaCl_2_, MnCl_2_, CuCl_2_, ZnCl_2,_ and FeCl_3_) on phosphatase activity, (r)OnTRAP5b was incubated at 37 °C for 30 min with a reaction mixture containing buffer (50 mM HAc-NaAc and different metal ions) with pH of 5.0 and 5 mM *p*NPP, and the reaction was stopped by adding 10 μL 1 M NaOH. The assay was performed independently three times. The phosphatase activity without metal ions was considered to be 100%, and the phosphatase activity determined under the remaining conditions was converted to relative phosphatase activity. To determine the effect of phosphatase-specific inhibitors (sodium tartrate and sodium fluoride) (Macklin, Shanghai, China) and EDTA were investigated as potential inhibitors of the (r)OnTRAP5b and (r)MBP. The phosphatase activity of (r)OnTRAP5b and (r)MBP was evaluated after 30 min of incubation at 37 °C with phosphatase-specific inhibitors or EDTA. The residual phosphatase activity was determined by adding 5 mM *p*NPP and incubating at 37 °C for 30 min. Then, 1 M NaOH (10 μL) was added to terminate the reaction, and the absorbance at 405 nm was measured using a microplate reader of multi-wavelength.

### 4.11. Effect of (r)OnTRAP5b on Immune-Related Genes

In order to investigate the effect of (r)OnTRAP5b on immune-related genes, three groups of MΦ were treated with (r)OnTRAP5b (100 μg/mL), (r)MBP (100 μg/mL) or PBS (as control). The MΦ were collected by centrifugation at 500× *g* for 10 min, followed by lysis with Trizol for RNA extraction. The mRNA levels of interleukin IL-1β (*IL-1β*), interleukin IL-6 (*IL-6*), interleukin IL-10 (*IL-10*), tumor necrosis factor-α (*TNF-α*), interleukin IL-8 (*IL-8*) and interleukin IL-12 (*IL-12*) were determined with Nile tilapia *β*-actin by the 2^−ΔΔCt^ method [43].

### 4.12. Measurement of Reactive Oxygen Species and Phagocytosis in MΦ

According to the manufacturer’s instructions, the Calcein AM/PI cell viability/cytotoxicity assay kit (Beyotime Biotech, Shanghai, China) was used to detect the viability of cells. The MΦ were adjusted to 1 × 10^6^ cells/mL with cell culture medium and then treated with (r)OnTRAP5b (100, 300, 500 μg/mL) or (r)MBP (100, 300, 500 μg/mL) at 25 °C for 12 h. After treatment, the cells were collected, and washed with ice-cold PBS, added 1 mL of Calcein AM/PI test working solution was to resuspend the cells and incubated at room temperature in the dark for 30 min. After incubation, the cells were placed on ice for testing. The fluorescence intensity was analyzed by BD LSRFortessa^TM^ (BD Biosciences, Franklin Lakes, NJ, USA).

Following the manufacturer’s instructions, the 2,7-dichlorofluorescin diacetate (DCFH-DA) ROS assay kit (Beyotime Biotech, Shanghai, China) was used to detect the ROS levels in MΦ. The MΦ were adjusted to 1 × 10^6^ cells/mL with cell culture medium and then treated with (r)OnTRAP5b (100 μg/mL, 500 μg/mL), (r)MBP (100 μg/mL, 500 μg/mL) or PBS (as control) at 25 °C for 6 h. After treatment, the cells were collected, and washed with ice-cold PBS, stained with 10 μM DCFH-DA in PBS, incubated in the dark at room temperature, and then cells were washed to remove the excess probe. The fluorescence intensity was analyzed by BD LSRFortessa^TM^ (BD Biosciences, NJ, USA).

The MΦ were resuspended and adjusted to 1 × 10^6^ cells/mL with cell culture medium and then treated with (r)OnTRAP5b or (r)MBP at a concentration of 100 and 500 μg/mL in cell culture at 25 °C for 6 h. After treatment, the MΦ were harvested and washed with ice-cold PBS by centrifugation at 100× *g*, 25 °C for 10 min. The cells were incubated with fluorescein isothiocyanate (FITC)-labeled *S. agalactiae* or FITC-*A. hyfrophila*. The cells and FITC-labeled *S. agalactiae* or FITC-*A. hyfrophila* were incubated in a dark environment for 4 h in a ratio of 1:20. After incubation the cells were centrifuged and washed with ice-cold PBS, and 0.25M D-sorbitol solution was added to remove FITC-labeled *S. agalactiae* or FITC-*A. hyfrophila* adhering to the cell surface. Finally, the collected and washed cells with ice-cold PBS, and then added 200 μL PBS to resuspend cells and analyzed by BD LSRFortessa^TM^ (BD Biosciences, NJ, USA). The phagocytosis rate was examined using the Flowjo V10 software.

### 4.13. Effects of Overexpression and Knockdown of OnTRAP5b on S. agalactiae Infection

Overexpression of OnTRAP5b in Nile tilapia was performed to explore its effects on *S. agalactiae* infection. The fish (16 ± 4 g) were randomly divided into three groups (15 fish in each group) and injected intramuscularly with 100 μL of pcOnTRAP5b (150 μg/mL), pcDNA3.1 (150 μg/mL) or PBS (control group). qRT-PCR was performed using qOnTRAP5b-F1/R1 to evaluate the level of OnTRAP5b in the liver, spleen, and head kidney (Table 1). Then Nile tilapia were infected with *S. agalactiae* (1 × 10^6^ CFU/mL). At 6, 12, and 24 h p.i, liver, spleen, and head kidney tissues were collected and weighed, with three fish randomly in each group at each time point. The number of active bacteria was calculated by the plate counting method.

This study evaluated the effect of OnTRAP5b on *S. agalactiae* infection. According to instructions, T7 RiboMAX™ Express RNAi System (Promega, Madison, WI, USA) kit was used to synthesize small interfering RNA (siRNA), which was utilized to silence the expression of OnTRAP5b in vivo. The primers dsOnTRAP5b-P1/P2 and dsOnTRAP5b-P3/P4, as well as dsGFP-P1/P2 and dsGFP-P3/P4 (Table 1), were designed for synthesizing the siRNA of dsOnTRAP5b and dsGFP. Nile tilapia (16 ± 4 g) were randomly separated into three groups, the experiment group was injected intramuscularly with 100 μL of dsOnTRAP5b (150 μg/mL), and the second and third groups were injected intramuscularly with 100 μL of dsGFP (150 μg/mL) or PBS (control group), respectively. After 12 h, qRT-PCR was performed using qOnTRAP5b-F1/R1 to determine the level of OnTRAP5b in the liver, spleen, and head kidney (Table 1). Then, the fish were injected with 100 μL of *S. agalactiae* (1 × 10^6^ CFU/mL). Bacterial colonies were examined using the plate smearing method after the liver, spleen, and head kidney were collected at 6, 12, and 24 h p.i.

### 4.14. Statistical Analysis

Excel 2019, GraphPad Prism 5, and Origin 8.0 were used to analyze statistical data. Data were presented as the mean ± standard deviation (SD), with statistical significance considered as a probability (*p*) value < 0.05. One asterisk (*) indicated a *p*-value between 0.01 and 0.05, and two asterisks (**) a *p*-value was < 0.01.

## Figures and Tables

**Table 1:** Primers used in this study.

Primer Name	Primer Sequence (5′ → 3′)	Usage
OnTRAP5b-F1	ATGGACAGGCTCATGTGTATAGTGT	Cloning of OnTRAP5b ORF sequence
OnTRAP5b-R1	CACTTTGCGTTTTGGCAGC	
OnTRAP5b-F2	CCGGATATCATGGTGGAACAAGGTGCCCT	Amplification of sequence of mature OnTRAP5b gene
OnTRAP5b-R2	CCGGGATCCCACTTTGCGTTTTGGCAGC	
OnTRAP5b-F3	CCGGGATCCGCCACCATGGACAGGCTCATGTGTATAGTGT	Overexpression of OnTRAP5b
OnTRAP5b-R3	CCGGATATCCACTTTGCGTTTTGGCAGC	
dsOnTRAP5b-P1	GGATCCTAATACGACTCACTATAGGTGTCTTCTCTCAGCCAT	Knockdown of OnTRAP5b
dsOnTRAP5b-P2	AAATGGCTGAGAGAAGACACCTATAGTGAGTCGTATTAGGATCC	
dsOnTRAP5b-P3	GGATCCTAATACGACTCACTATAATGGCTGAGAGAAGACACC	
dsOnTRAP5b-P4	AAGGTGTCTTCTCTCAGCCATTATAGTGAGTCGTATTAGGATCC	
dsGFP-P1	GGATCCTAATACGACTCACTATAGCCACAACGTCTATATCAT	Synthetic GFP siRNA
dsGFP-P2	AAATGATATAGACGTTGTGGCTATAGTGAGTCGTATTAGGATCC	
dsGFP-P3	GGATCCTAATACGACTCACTATAATGATATAGACGTTGTGGC	
dsGFP-P4	AAGCCACAACGTCTATATCATTATAGTGAGTCGTATTAGGATCC	
qOnTRAP5b-F1	ACTTCTATTTTAGCGGCGTCAA	qRT-PCR of OnTRAP5b gene
qOnTRAP5b-R1	GCACCACCGTATCAATCATCA	
qIL-1β-F	CGTGCCAACAGTGAGAAAGCG	qRT-PCR analysis
qIL-1β-R	CAGGAGGGACGGAAGGGAT	
qIL-6-F	ACAGAGGAGGCGGAGATG	
qIL-6-R	GCAGTGCTTCGGGATAGAG	
qIL-10-F	TGGAGGGCTTCCCCGTCAG	
qIL-10-R	CTGTCGGCAGAACCGTGTCC	
qTNFα-F	GCTGAGGCTCCTGGACAAAA	
qTNFα-R	TCTGCCATTCCACTGAGGTCTT	
qIL-8-F	GATAAGCAACAGAATCATTGTCAGC	
qIL-8-R	CCTCGCAGTGGGAGTTGG	
qIL-12-F	GTGGTCTTGGTAAAGGCCGA	
qIL-12-R	AGTTTTGCCGGTCTCACGAT	
qβ-actin-F	CGAGAGGGAAATCGTGCGTGACA	
qβ-actin-R	AGGAAGGAAGGCTGGAAGAGGGC	

## Data Availability

The data presented in this study are available from the corresponding author on reasonable request.

## Supplementary Materials

The following supporting information can be downloaded at: https://www.mdpi.com/article/10.3390/ijms24087179/s1.

## Abbreviations

BHI	Brain heart infusion
CFU	Colony-Forming Units
DCFH-DA	2,7-dichlorofluorescin diacetate
DCs	Dendritic cells
EDTA	Ethylene Diamine Tetraacetic Acid
FBS	Fetal bovine serum
FITC	Fluorescein isothiocyanate
h p.i	Hour post infection
IL-1β	Interleukin IL-1β
IL-6	Interleukin IL-6
IL-8	Interleukin IL-8
IL-10	Interleukin IL-10
IL-12	Interleukin IL-12
IPTG	Isopropyl-β-D-thiogalactopyranoside
LB	Luria-Bertani
LPS	Lipopolysaccharide
MPP	Metallophosphatase
MΦ	Macrophages
NCBI	National Center for Biotechnology Information
O.D.	Optical density
OnTRAP5b	Oreochromis niloticus TRAP5b
ORF	Open reading frame
P/S	Penicillin G and Streptomycin
PBS	Phosphate buffer saline
PCR	Polymerase Chain Reaction
pNPP	p-nitrophenyl phosphate
qRT-PCR	Quantitative real-time PCR
RNAi	RNA interference
(r)OnTRAP5b	Recombinant protein OnTRAP5b
ROS	Reactive oxygen species
RPMI-1640	Roswell Park Memorial Institute 1640 medium
SD	Standard deviation
SDS-PAGE	SDS-polyacrylamide gel electrophoresis
siRNA	Synthesize small interfering RNA
TNF-α	Tumor necrosis factor-α
TRAP5	Tartrate-resistant acid phosphatase type 5
