# Supplementary material for: Identification and Functional Analysis of Tartrate-Resistant Acid Phosphatase Type 5b (TRAP5b) in Oreochromis niloticus
Source: Int J Mol Sci. 2023 Apr 13;24(8):7179. doi: 10.3390/ijms24087179 (PMC10138680; doi:10.3390/ijms24087179)
Supplement: Supplementary file 1 [file ijms-24-07179-s001.zip › ijms-2311951-supplementary.pdf]

International Journal of
Molecular Sciences

Supplementary materials

Identification and Functional Analysis of Tartrate-Resistant Acid Phosphatase Type 5b (TRAP5b) in *Oreochromis niloticus*

Yang Lei [1], Shengli Fu [1,2], Yanjian Yang [1], Jianlin Chen [1,†], Bingxi Li [1,†], Zheng Guo [1,*] and Jianmin Ye [1,2,*]

[1] Guangzhou Key Laboratory of Subtropical Biodiversity and Biomonitoring, Institute of Modern Aquaculture Science and Engineering, School of Life Sciences, South China Normal University, Guangzhou 510631, China
[2] Guangdong Provincial Engineering Technology Research Center for Environmentally-Friendly Aquaculture, South China Normal University, Guangzhou 510631, China
* Correspondence: 20151053@m.scnu.edu.cn (Z.G.); jmye@m.scnu.edu.cn (J.Y.)
† Current address: Guangdong Provincial Engineering Research Center for Aquatic Animal Health Assessment, Shenzhen Public Service Platform for Evaluation of Marine Economic Animal Seedings, Shenzhen Institute of Guangdong Ocean University, Shenzhen 518120, China.

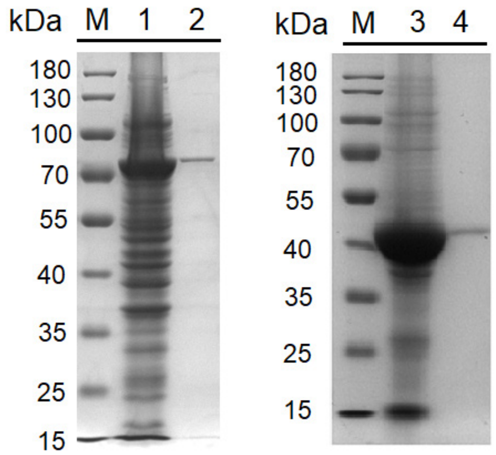

Figure S1. SDS-PAGE analysis of prokaryotic recombinant expressed and purified (r)OnTRAP5b and (r)MBP. Lane M, protein molecular weight marker; Lane 1, unpurified (r)OnTRAP5b protein; Lane 2, purified (r)OnTRAP5b protein; Lane 3, unpurified (r)MBP protein; Lane 4, purified (r)MBP protein.

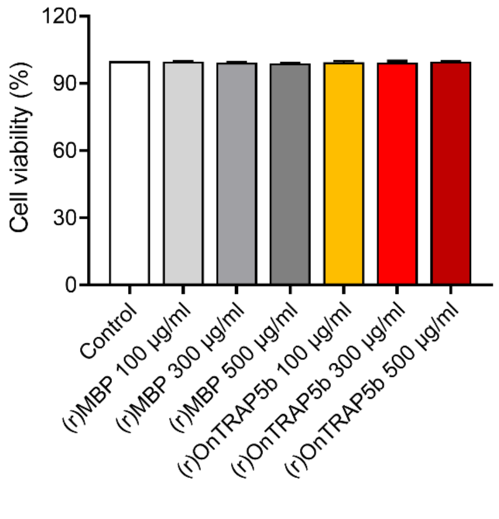

Figure S2. The effect of (r)OnTRAP5b on macrophages viability. Statistical analysis of macrophage viability after stimulation with (r)OnTRAP5b. Three biological replicates were performed and the date are presented as means ± SD (n = 3). ** $p < 0.01$, * $p < 0.05$.

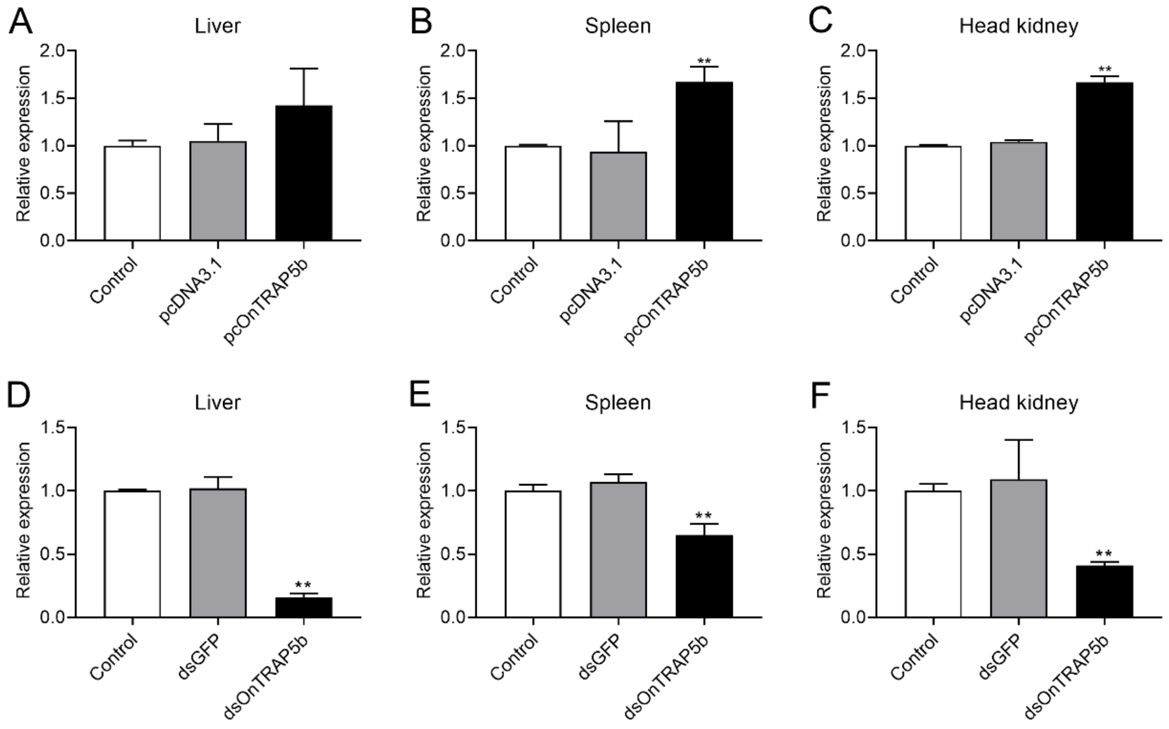

Figure S3. Analysis of the mRNA level of OnTRAP5b after overexpression and knockdown *in vivo*. Nile tilapia were administered with pcOnTRAP5b, pcDNA3.1 or PBS (control) and *OnTRAP5b* expression in liver (A), spleen (B) and head kidney (C) was determined by qRT-PCR for 5 d. Nile tilapia were administered with dsOnTRAP5b, dsGFP or PBS (control) and *OnTRAP5b* expression in liver (D), spleen (E) and head kidney (F) was determined by qRT-PCR for 12 h. The expression level of the control fish was set as 1. The values are shown as means ± SD ($n = 3$), N, the number of times the experiments were performed. ** $p < 0.01$, * $p < 0.05$.

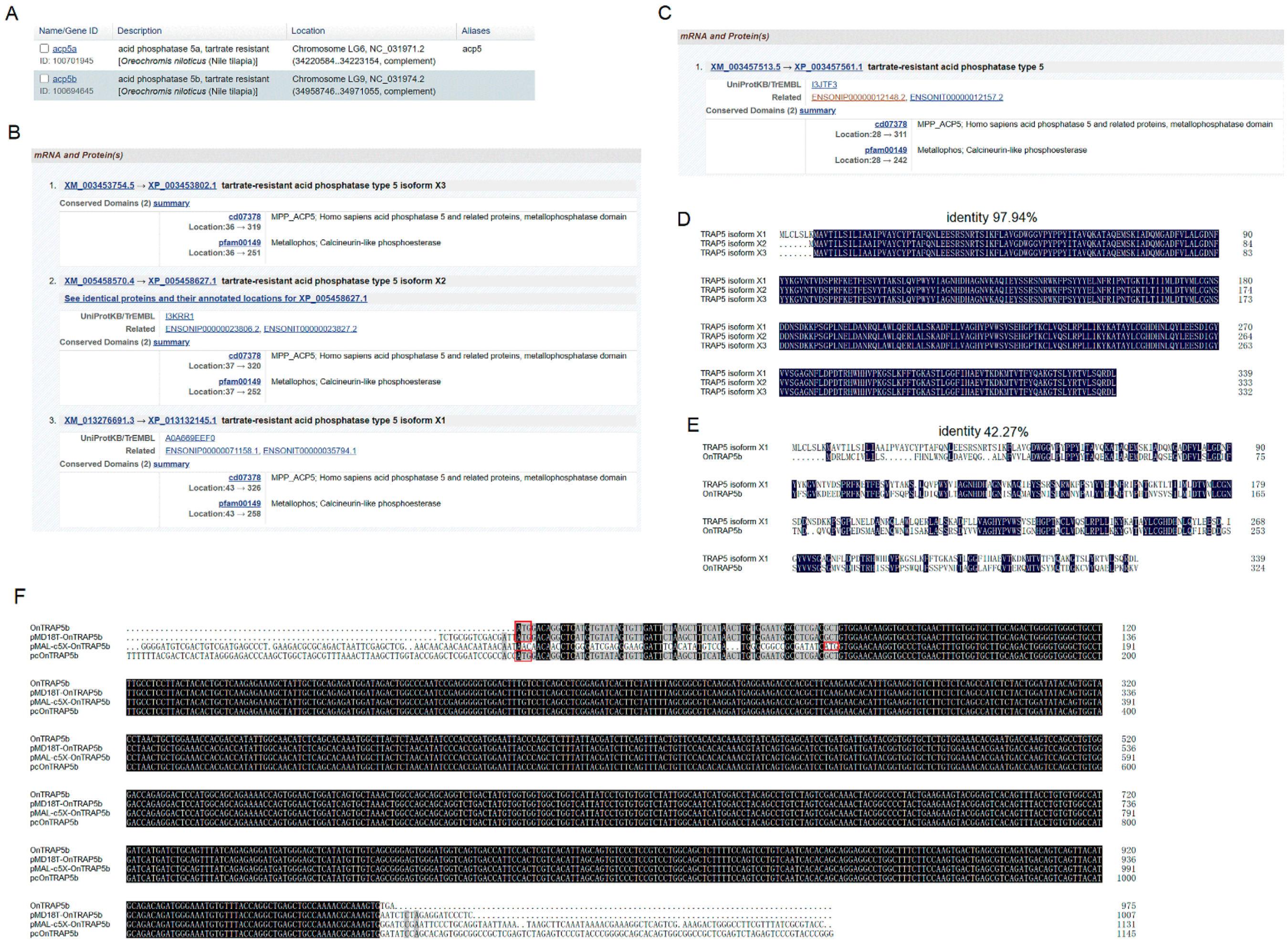

Figure S4 Sequence alignment analysis of TRAP5a and TRAP5b and sequencing analysis of plasmid sequences in Nile tilapia. (A) Two types of tartrate-resistant acid phosphatase gene sequences (ACP5a also named TRAP5a, ACP5b also named TRAP5b). (B/C) ACP5a occurs as three distinct isoforms, namely TRAP5 isoform X1, TRAP5 isoform X2 and TRAP5 isoform X3. ACP5b has no isoforms and the mRNA sequence of ACP5b is unique. (D) Sequence alignment analysis of the three isoforms of ACP5a showed a similarity of 97.94%. (E) Sequence alignment analysis of the TRAP5 isoform X1 and TRAP5b showed a similarity of 42.27%. (F) The plasmid sequences encoding OnTRAP5b (pMD18T-OnTRAP5b, pMAL-c5X-OnTRAP5b, and pcOn-TRAP5b) have been confirmed as the OnTRAP5b sequence in the Nile tilapia transcriptome database by sequencing analysis. The start codon (ATG) was circled with a red box.
